# NGF eye-drops topical administration in patients with retinitis pigmentosa, a pilot study

**DOI:** 10.1186/s12967-015-0750-3

**Published:** 2016-01-09

**Authors:** Benedetto Falsini, Giancarlo Iarossi, Antonio Chiaretti, Antonio Ruggiero, Manni Luigi, Lucia Galli-Resta, Giovanni Corbo, Edoardo Abed

**Affiliations:** Institute of Ophthalmology, Policlinico Gemelli, Catholic University of Sacro Cuore, Lgo F. Vito 1, 00168 Rome, Italy; Ophthalmology Department, Ospedale “Bambin Gesù”, 00165 Rome, Italy; Translational Pharmacology, CNR, Rome, Italy; Institute of Pediatrics, Policlinico Gemelli, Catholic University, 00168 Rome, Italy; Neuroscience Institute, CNR, 56124 Pisa, Italy

**Keywords:** Retinitis pigmentosa, Nerve growth factor, Eye drops, Photoreceptor, Focal electroretinogram, Visual field

## Abstract

**Background:**

Preclinical trials have shown beneficial effects of nerve growth factor (NGF) administration on visual function in animal models of retinitis pigmentosa (RP). The aim of this pilot study was to explore the potential efficacy of short term NGF eye drops treatment in patients affected by RP.

**Methods:**

The trial consisted in 10 days daily administration of murine NGF as eye-drops for a total dose of 1 mg NGF/pt. Eight RP patients at an advanced stage of the disease were included in the trial. To monitor safety and potential adverse effects subjects underwent standard clinical measures and were requested to report any general or topic alterations following NGF assumption. Retinal function was assessed at baseline and after treatment by best-corrected visual acuity measurement (BCVA), macular focal electroretinogram (fERG) recording and Goldmann visual field testing.

**Results:**

A transient tolerable local corneal irritation was the only adverse effect reported. fERG and BCVA remained within the limits determined by test–retest analysis of a large cohort of RP patients. Three patients reported a subjective feeling of improved visual performance. This was associated to a temporary enlargement of the visual field in all three patients and to improved fERG in two of the three.

**Conclusions:**

Short-term administration of NGF eye-drops caused neither significant adverse effects nor visual function losses in the tested RP patients. A minority of patients experienced an improvement of visual performance as shown by Goldmann visual field and fERG. This study supports the safety and possible efficacy of NGF eye-drops administration in RP patients.

Trial registration: EudraCT n. 2008-004561-26

## Background

Inherited retinal dystrophies of photoreceptors such as retinitis pigmentosa (RP) are an important cause of severe vision loss.

There is presently no cure for RP, but considerable effort is devoted to the search of rescue strategies. Even if the exact mechanisms leading to photoreceptor death in the various phenotypes of retinal degeneration are not fully understood, photoreceptor apoptosis is considered to be the final common event in the disease process [1–3]. Because of their ability to inhibit the apoptotic cascade, neurotrophic factors may represent a promising therapeutic strategy in RP. This was first demonstrated by slowing down progressive photoreceptors loss in Royal College of Surgeons (RCS) rats following intravitreal injection of basic fibroblast growth factor (bFGF) [4].

The neurotrophins (NTs) are a family of peptide growth factors homologous to Nerve Growth Factor (NGF) that regulate the development, differentiation, survival and function of neuronal cells. There is evidence that exogenous NT administration promotes photoreceptor survival in animal models of both light induced [5–9] and inherited retinal degeneration [8, 10–12].

Among NTs, NGF seems particularly promising for testing. Its exogenous administration has been shown to promote photoreceptor survival in animal models of RP [10, 11].

Furthermore, preclinical evidence in rodents shows significant availability of this NT in the retina following its administration as eyedrops [13]. Indeed, clinical studies also suggest NGF eyedrops biological activity in the visual system of patients with glaucomatous optic neuropathy [14] and childhood optic glioma [15, 16].

One possible limitation of the use of neurotrophic factors is their potential negative effect on photoreceptor function, as a result of downregulation of phototransduction cascade enzymes. It is indeed known, from both preclinical [17] and clinical [18] studies, that the NTs bFGF and ciliary neurotrophic factor (CNTF) may depress retinal sensitivity as shown by electroretinogram [17] and visual field examination [18]. Thus a functional negative effect may represent the price to be paid to obtain photoreceptor neuroprotection.

The aim of this investigation was to investigate the potential efficacy and adverse effects of murine NGF eyedrops administration in RP patients.

## Methods

The present pilot study (EudraCT n. 2008-004561-26) followed the tenets of the Declaration of Helsinki and was approved by the ethics committee of the institution. All the enrolled patients were fully informed as to the nature and goals of the study. Written informed consent was obtained from all patients.

### Patient recruitment and Inclusion criteria

Sixteen eyes of 8 patients (6 males, 2 females; average age 49.7 ± 14.3 years) affected by RP were included in the study (Table 1). All patients had progressive forms of RP based on history, clinical findings and ERG abnormalities. Furthermore, patients met the following inclusion criteria: (1) typical RP with a rod-cone pattern of retinal dysfunction, as determined by standard Ganzfeld electroretinography, dark-adapted Tuebinger perimetry, and classic fundus appearance. (2) Advances stage of the disease (at baseline: central portion of visual field with Goldman V/4e <15 deg; fERG <1 uV). (3) Known inheritance pattern and/or genotype under study. (4) At least 1 years of fERG and clinical examination follow-up, with a minimum of three visits. (5) No or minimal ocular media opacities. (6) No concomitant ocular (e.g. glaucoma, amblyopia) or systemic diseases. Patients with non- Usher syndromic sub-types of RP, Leber’s congenital amaurosis or early onset RP with atypical functional patterns were excluded.

**Table 1:** Patient details

Patient#	Gender	Age	Inheritance	Genetic mutation	BCVA^a^ (decimal)	fERG^a^ (mV)
1	M	38	Autosomal recessive	CERKL R257X (homozygous)	0.39	0.30
2	M	28	Autosomal recessive	Unknown	0.17	0.09
3	M	59	Autosomal dominant	Unknown	0.85	0.58
4	M	69	Autosomal recessive	CRX V242M (heterozygous)	0.32	0.12
5	F	48	Autosomal dominant	RHO R135W	0.10	0.41
6	F	62	Autosomal recessive	Unknown	0.05	0.30
7	M	57	Autosomal dominant	Unknown	0.57	0.16
8	M	37	X-linked	Unknown	0.10	0.27

^a^Averaged between eyes

### Measures of ocular function and electroretinography

A full general and ophthalmologic examination (including detailed family history, anterior segment biomicroscopy, BCVA, direct and indirect ophthalmoscopy, intraocular pressure measurement) was performed on each patient at baseline.

Best-corrected visual acuities were obtained with a projected Snellen chart. Kinetic visual fields were measured to the V4e white test light of the Goldmann perimeter against the standard background of 31.5 apostilbs. Goldmann visual fields were digitized and total visual field areas were calculated.

Cone focal ERGs (fERG) were recorded from the central 18° region using a uniform red field superimposed on an equiluminant steady adapting background, used to minimize stray-light modulation [19, 20]. The stimulus was generated by a circular array of eight red LEDs (λ maximum, 660 nm; mean luminance, 93 cd/m^2^) presented on the rear of a Ganzfeld bowl (white-adapting background). A diffusing filter in front of the LED array made it appear as a circle of uniform red light. fERGs were recorded in response to the sinusoidal 95 % luminance modulation of the central red field. Flickering frequency was 41 Hz. Patients fixated monocularly at a 0.25° central fixation mark, under the constant monitoring of an external observer. Pupils were pharmacologically (1 % tropicamide and 2.5 % phenylephrine hydrochloride) dilated to a diameter ≥8 mm, and all subjects underwent a pre-adaptation period of 20 min to the stimulus mean luminance. fERGs were recorded by an Ag–AgCl electrode taped on the skin over the lower eyelid. A similar electrode, placed over the eyelid of the contralateral patched eye, was used as reference (inter-ocular recording). fERG signals were amplified (10^6^-fold), bandpass filtered between 1 and 100 Hz (6 dB/oct), and averaged (12-bit resolution, 2-kHz sampling rate, 200–600 repetitions in 2–6 blocks). Off-line discrete Fourier analysis quantified the amplitude and phase lag of the response fundamental harmonic (1st harmonic) at 41 Hz.

### Ocular and systemic complications potentially related to ngf administration

During the entire period of assessment (40 days; see below) particular attention was paid to detect ocular and/or systemic side effects. Potential ocular complications included inflammation (external or uveitis), pain, development of lens opacities, and increased intraocular pressure. Systemic complications previously reported in the literature include allergic reactions, systemic pain as well as weight loss [21].

A comprehensive medical evaluation was carried out by a general physician at day zero, and at the end of the NGF treatment. All patients received oral and written information about the experiment procedures before signing the informed consent.

### Nerve growth factor isolation

NGF (2.5S) was purified from male mouse submandibular glands as already described [15, 22]. Briefly, the extract of submandibular glands of adult male mice was passed through subsequent cellulose columns, to separate NGF by adsorption. NGF-containing fractions were analyzed by spectrophotometry and Western blot analysis. NGF purity (>95 %) was estimated by high-performance liquid chromatography, while its biological activity was evaluated by neurite outgrowth stimulation in rat PC12 cells. Purified NGF was dialyzed, lyophilized under sterile conditions, and stored at −20 °C until used. At the time of use, purified NGF was dissolved in 0.9 % sterile saline solution in concentrations of 200 µg/mL. The concentration of NGF in this solution was stable over the 10 day treatment time.

### NGF administration schedule

A total of 1 mg of NGF diluted in 5 mL of saline solution was administered in the form of eye drops onto the conjunctiva of both eyes for 10 consecutive days 3 times a day. This amount is considered sufficient to reach and stimulate NGF receptors in most cerebral cholinergic areas of the brain and optic pathways, as previously reported [13]. We preferred to use murine NGF, instead of human-recombinant NGF, because contrasting results have been reported on the efficacy of the latter, mainly due to a lack of in vivo studies [16].

### Testing schedule

fERG examinations were performed at baseline, at the end of the 10 days period of NGF administration and 30 days later. BCVA measurement and Goldmann visual field examination were performed at baseline and 30 days after the end of NGF administration.

### Data analysis

Changes in BCVA and fERG amplitude obtained after treatment were evaluated as individual changes, as well as in the contest of test–retest variability data obtained from a large cohort of RP patients followed clinically at the Visual Electrophysiology Service of the Institute of Ophthalmology at Universita’ Cattolica del S. Cuore, which have been subject of a long term follow-up study [23].

In each patient, the pre and post treatment total areas of Goldmann visual fields were compared. A percentage difference >20 % was considered clinically significant according to previous studies on test–retest variability in patients with RP [24].

## Results

Both clinical and subjective reports showed that none of the patients suffered any adverse reaction, except for mild and transient conjunctival hyperemia and ocular pain, as already reported in previous studies administering NGF eye drops [25].

**Fig. 1:**
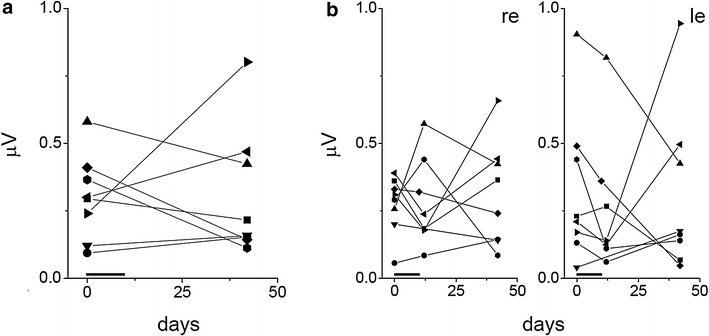
Macular focal ERG (fERG) time course in NGF treated RP patients. Individual patient fERGs averaged between the two eyes are shown in (**a**), single eye data are shown in (**b**). For the sake of simplicity data collected soon after the end of treatment (10 days) are only shown in (**b**). Each patient is identified by the same symbol in (**a**, **b**). Patient-symbol association is indicated below the *graph*s

Individual macular focal ERG (fERG) time courses over the trial are shown in Fig. 1. Figure 1a summarizes the results, showing individual patient data averaged between the two eyes at baseline and at the end of the trial. Individual eye data before, at end of the 10 days NGF treatment and 30 days afterwards are shown for each patient in Fig. 1b. The same symbol-patient association is used in all Figures to allow identification of each case.

**Fig. 2:**
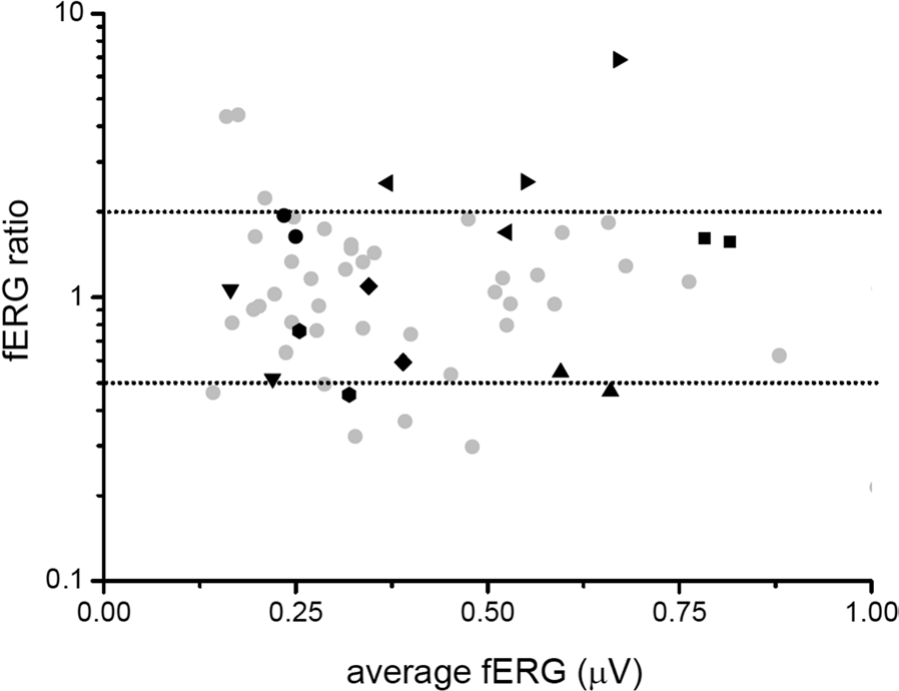
Macular focal ERG (fERG) changes following NGF treatment in the context of RP patient test–retest fERG variability. *Black symbols* correspond to the NGF treated patient (same patient-symbol association as in Fig. 1), *gray circles* are test–retest data from long term follow-up RP patients from the clinic database (N = 94 eyes from 47 patients). *Dotted horizontal lines* represent the 10 and 90th percentile of the test–retest data sample. Average test–retest time span 130 ± 76 days. Each patient is identified by the same symbol as in Fig. 1

fERG changes over this period of time are heterogeneous among patients, with both improvements and decrements. To place these measures in context, we plotted the overall fERG changes in each patient eye from baseline to end of trial against the background of test–retest variability in fERG measures observed in a large database of RP patients followed by our Ophthalmology Clinic (Fig. 2). This comparison showed that all but 2 cases were within the 10–90 percentiles limits of test–retest variability for fERG measures in RP patients. Indeed, when considered as a population, fERG changes over the trial period that did not statistically differ from the fERG changes observed in the test–retest variability data in the above mentioned RP patient database (2-sample Student *t* test P = 0.48 N = 8 present trial; N = 47 test–retest fERG data from RP database). Interestingly, however, the two trial cases showing a larger improvement than variability cut-offs also reported subjective improvement of visual performance (see below).

**Fig. 3:**
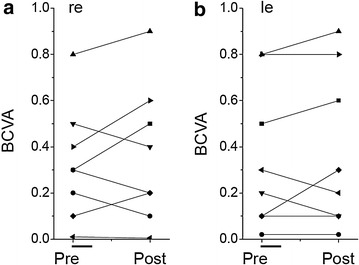
Best corrected visual acuity (BCVA) time course in NGF treated RP patients. Individual patient BCVA in either eye are shown before and 30 days after the end of NGF treatment. Each patient is identified by the same symbol as in Fig. 1

Individual best corrected visual acuity (BCVA) measures at baseline and 30 days after the end of NGF treatment are shown in Fig. 3a, b for either eye of the eight trial patients.

**Fig. 4:**
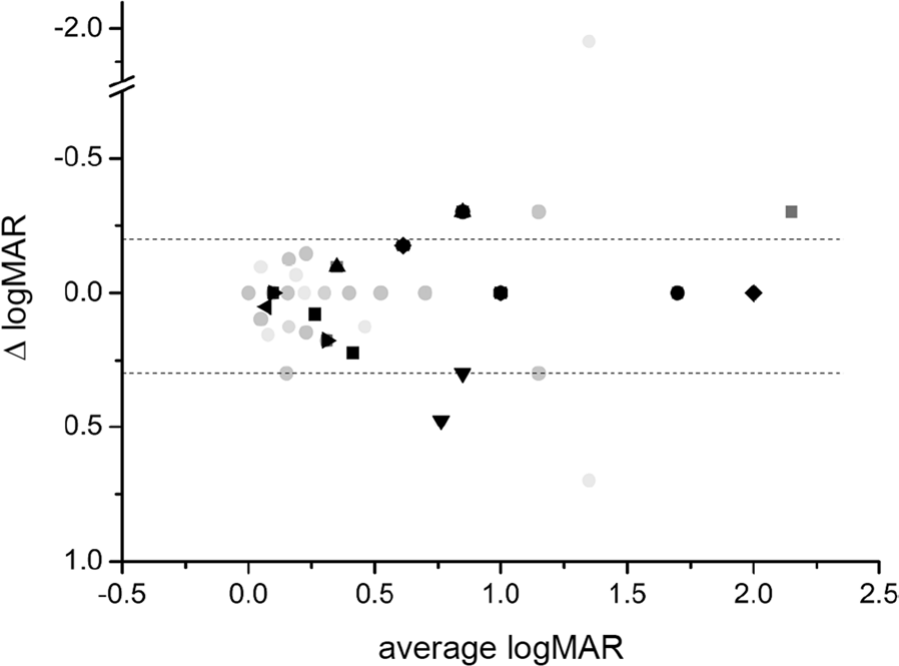
Best corrected visual acuity (BCVA) changes following NGF treatment in the context of RP patient test–retest BCVA variability. *Black symbols* correspond to the NGF treated patient (same patient-symbol association as in Fig. 1), *gray circles* are test–retest data available from long term follow-up RP patients in the clinic database (N = 35 eyes from 18 patients). *Dotted horizontal lines* represent the 10 and 90th percentile of the test–retest data sample. Average test–retest time span 130 ± 71 days. Each patient is identified by the same symbol as in Fig. 1

BCVA changes over this period of time were heterogeneous among patients, but remained within the limits of BCVA test–retest variability in the database of RP patients followed for over 4 years by our Ophthalmology Clinic (Fig. 4). When measuring BCVA relative changes as the ratio between Delta(logMAR) and average(logMAR) before and at the end of treatment, the difference between BCVA variation over trial and test–retest variability in RP patients was not statistically significant (Student t-test, P = 0,7157, N = 16 eyes from 8 NGF treated eyes; 35 eyes from 18 RP database patients).

**Fig. 5:**
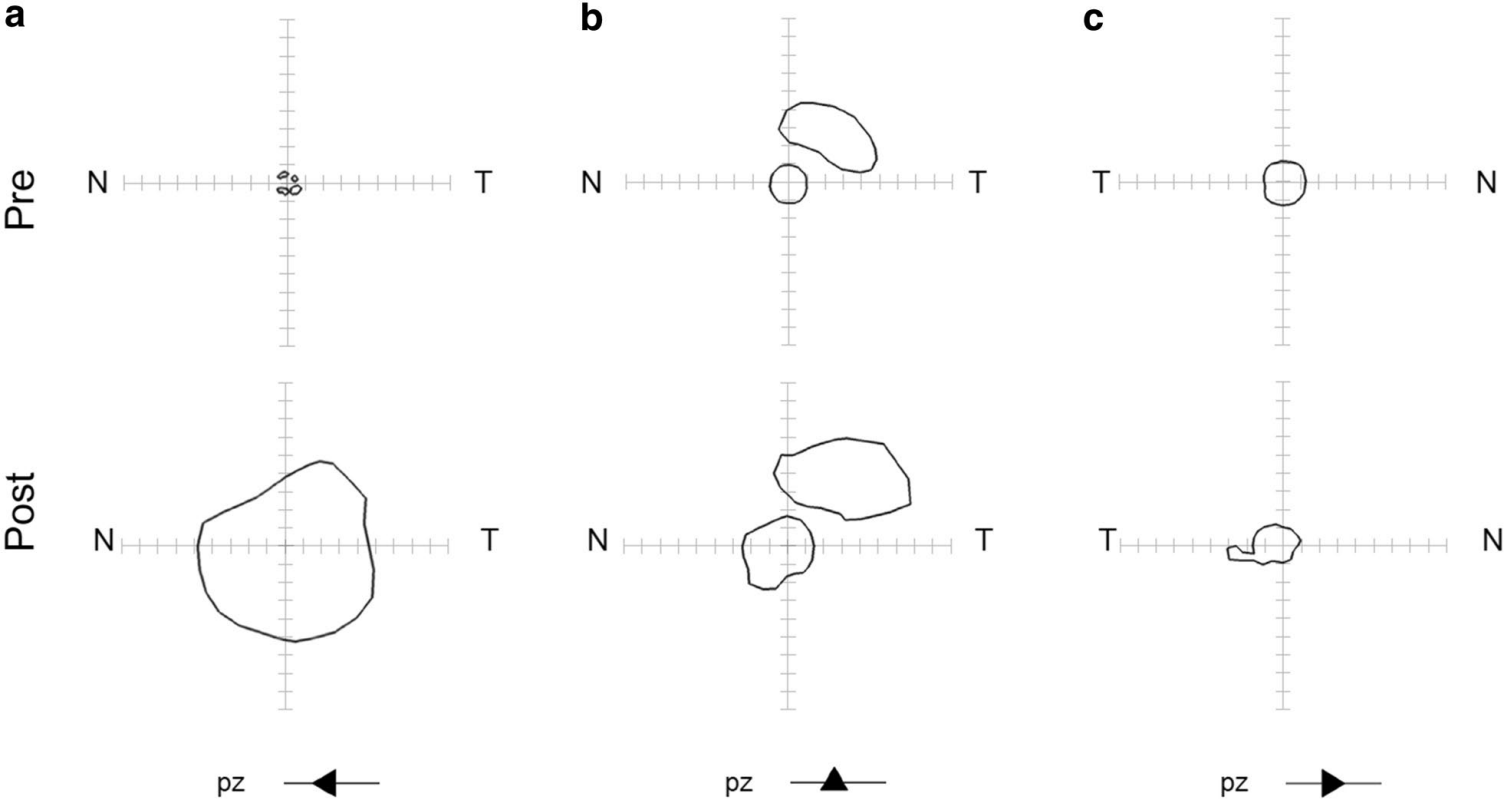
Goldmann kinetic visual fields obtained 1 year before and 30 days after NGF treatment in the three trial patients (**a**–**c**) that subjectively reported improved visual function in the trial. Goldmann visual fields were obtained with the V/4e white light stimulus. Pre and post measurements were obtained in the same experimental setting. *T* temporal; *N* nasal. Patient—symbol association is the same as in Fig. 1

After treatment, the area of Goldmann V4e isopter significantly (>20 %) increased in 3 patients (37.5 %), as shown in Fig. 5, and was unchanged in the remaining 5 patients (62.5 %). None of the patients had worsening of kinetic visual field. Interestingly, all patients with visual field enlargement reported subjective improvement of visual performance and two of three patients showed an increase of fERG amplitude beyond test–retest variability 90th percentile.

## Discussion

The present pilot study was designed to explore the potential neuroprotective effect of NGF eye-drops in patients affected by RP.

No significant general adverse effects were clinically recorded nor reported by any patient. Only minor ocular side effects such as conjunctival hyperemia and ocular pain were reported, in line with previous studies using NGF eye-drops in other ocular pathologies [25].

Furthermore, no adverse changes in central retinal function were found in either BCVA or macular cone flicker ERG recorded with a specialized sub-microvolt technique developed in our laboratory, nor reported subjectively by the subjects. This is particularly relevant considering that previous studies based on the same rationale of testing neurotrophins for neuroprotection, found reduced central retinal sensitivity in most study eyes receiving implants of the neurotrophin CNTF [18].

Interestingly, three patients experienced a subjective improvement of visual performance confirmed by a significant enlargement of Goldmann visual field and, in two cases, by an increase of macular cone function as determined by fERG. This data apparently indicates that NGF eye drops administration may improve retinal function at least in some cases with RP suggesting that NGF may exert a neuroprotective and/or neuro-enhancement effect in these patients. The neuroprotective effect of NGF on photoreceptor cells has been already demonstrated in animal models of RP. In 1996, Lambiase and Aloe showed that intravitreal and retrobulbar injection of NGF caused a significant delay of retinal degeneration with preservation of the outer nuclear layer (ONL) in C3H mice10.

Additionally, Lenzi et al [11] reported that retrobulbar injection of NGF reduced ONL thinning in RCS rats.

The exact mechanism responsible for the neuroprotective effect of NGF on photoreceptors is still unknown. There is a large body of evidence demonstrating that, in the mammalian retina [26–29], NGF receptors are expressed in Müller and retinal ganglion cells (RGC) but not in the ONL suggesting that the neuroprotective effect of NGF on photoreceptor cells may be probably indirect. As already suggested by Whalin et al. [30, 31], neurotrophins may bind their receptors on Müller cells and increase the release of multiple growth factors that may act on photoreceptor cells. This hypothesis seems to be confirmed by the finding that, in RCS rats [11], retrobulbar administration of NGF enhanced the expression of multiple growth factors including brain-derived neurotrophic factor (BDNF), FGF, transforming growth factor beta (TGF-β), vascular endothelial growth factor (VEGF) and neuropeptide-Y (NPY). Furthermore, it has been demonstrated that NGF may increase outer retina oxygenation by modulating the expression of VEGF in the RGC layer [32]. As suggested by Abed et al [33], the increase of oxygen tension may prevent photoreceptors apoptosis in specific models and stages of retinal degeneration.

A limitation of the present study is the limited number of patients, which however is not uncommon in this type of studies, where ethical reasons tend to oppose the use of large samples of patients to test safety. Furthermore, the absence of a placebo-treated control group and the short duration of follow-up may reduce the reliability of these fascinating results.

## Conclusions

In conclusion, this study showed that NGF eye drops administration is well tolerated without significant adverse effects. In a minority of patients, the treatment was associated with a subjective improvement of visual function confirmed by Goldmann visual field and fERG supporting the feasibility of a randomized clinical trial, with an estimate of the potential effect size, testing the neuroprotective efficacy of NGF eye-drop treatment in RP patients.
