# Erratum to: NGF eye-drops topical administration in patients with retinitis pigmentosa, a pilot study

**DOI:** 10.1186/s12967-016-0800-5

**Published:** 2016-02-08

**Authors:** Benedetto Falsini, Giancarlo Iarossi, Antonio Chiaretti, Antonio Ruggiero, Luigi Manni, Lucia Galli-Resta, Giovanni Corbo, Edoardo Abed

**Affiliations:** Institute of Ophthalmology, Policlinico Gemelli, Catholic University of Sacro Cuore, Lgo F. Vito 1, 00168 Rome, Italy; Ophthalmology Department, Ospedale “Bambin Gesù”, 00165 Rome, Italy; Institute of Translational Pharmacology, CNR, Rome, Italy; Institute of Pediatrics, Policlinico Gemelli, Catholic University, 00168 Rome, Italy; Neuroscience Institute, CNR, 56124 Pisa, Italy

## Erratum to: J Transl Med (2016) 14:8 DOI 10.1186/s12967-015-0750-3

The original version of this article [1] unfortunately contained a mistake. In the author list, the given and family names of author Luigi Manni were reversed. The original article has now been updated with the names in the correct order. The affiliations of authors Chiaretti, Ruggiero and Manni have also been updated.
